# Phylogeny and biogeography of Sumatra´s cloud forest lizards of the genus *Dendragama* and status of *Acanthosaura schneideri*

**DOI:** 10.3897/zookeys.995.49355

**Published:** 2020-11-18

**Authors:** Kyle J. Shaney, Michael B. Harvey, Amir Hamidy, Nia Kurniawan, Eric N. Smith

**Affiliations:** 1 Departamento de Ecología de la Biodiversidad, Instituto de Ecología, Universidad Nacional Autónoma de México, Ap. Postal 70-275, Ciudad Universitaria, Ciudad de México, 04510, México University of Texas Arlington United States of America; 2 Laboratory of Herpetology; Museum Zoologicum Bogoriense; Research Center for Biology, Indonesian Institute of Sciences–LIPI; Jl. Raya Jakarta Bogor km 46; Cibinong, west Java, 16911, Indonesia Universidad Nacional Autónoma de México Ciudad de México Mexico; 3 Department of Biology; Universitas Brawijaya; Jl. Veteran; Malang, East Java, 65145, Indonesia Broward College Davie United States of America; 4 Department of Biological Sciences; Broward College; 3501 S.W. Davie Road; Davie, FL 33314, USA Indonesian Institute of Sciences Bogor Indonesia; 5 The Amphibian and Reptile Diversity Research Center and Department of Biology; University of Texas at Arlington; 501 S. Nedderman Drive; Arlington, TX 76010; 775-351-5277, USA Universitas Brawijaya Malang Indonesia

**Keywords:** Barisan Range, biodiversity, Indonesia, IUCN, Pacific Ring of Fire, phylogeography, Toba eruption

## Abstract

Lizards of the genus *Dendragama* are endemic to the highland cloud forests of Sumatra’s Barisan Mountain Range in western Indonesia, and recent studies have uncovered widespread diversity within the genus. Here, a suite of morphological characters and mitochondrial DNA are used to compare three geographically isolated populations of *D. boulengeri* from (1) Mount Kerinci in Jambi province, (2) Mount Marapi of west Sumatra, and (3) the Karo Highlands of north Sumatra. Additional phylogeographic analyses with two recently described sister species, *D. australis* and *D. dioidema* were conducted. Five genetically distinct clades of *Dendragama*, all distributed allopatrically of one another were identified and some are suspected to inhabit small distributions. Morphological and genetic data confirm the Karo Highlands population *D. schneideri* (previously *Acanthosaura schneideri* Ahl, 1926) should be revalidated from the synonymy of *D. boulengeri*. *Dendragama schneideri* is endemic to montane forests of the Karo Highlands surrounding Lake Toba in Sumatra Utara province. Pairwise genetic distances of 6–11% separate *D. schneideri* from congeners. Two distinct clades of *D. boulengeri* from Mount Kerinci and Mount Marapi were identified, which are 5.0% genetically distant from one another. Using morphological characters, we provide the first key for distinguishing between species of *Dendragama*. Based on biogeographic patterns and levels of genetic variation it is suspected that at least 18 other isolated cloud forest locations may hold new species or divergent populations of *Dendragama* but lack survey work. Collectively, these comparisons among populations of montane lizards further elucidate the complex biogeographic history of Sumatra’s montane forest species and the first phylogeny of the genus *Dendragama*.

## Introduction

Uncovering tropical diversity is essential for conservation initiatives and understanding complex ecological and evolutionary processes (Ladle and Whittaker 2011). However, many regions and taxonomic groups across the globe remain largely unstudied, and for a multitude of reasons, Indonesia is particularly under-represented in this regard (O’Connell et al. 2018). Sumatra’s Barisan Mountains are especially interesting because the entirety of the range is a volcanically active strip of the “Pacific Ring of Fire” which runs along the edge of Sumatra’s west coast.

Volcanic activity and other historical biogeographic pressures throughout the Barisan Range have led to the development of a fascinating array of biodiversity (Demos et al. 2016), including a whole suite of montane forest species that live in high elevation cloud forest (temperate moist forest typically between 1,300 and 2,800 m of elevation in the tropics). However, Sumatra’s cloud forest biodiversity has remained largely unexplored and the pressures that have driven the development of that diversity are not yet fully understood (O’Connell et al. 2018). Reptilian diversity is no exception in this regard despite the fact that Sumatra is known to harbor some of the most evolutionarily unique lineages of reptiles, including enigmatic species like the Modigliani’s nose-horned lizard which was recently rediscovered (Putra et al. 2020).

Interestingly, despite the lack of herpetofaunal survey work throughout the region, Sumatra is already considered the most draconine (family Agamidae) diverse island in southeast Asia (Harvey et al. 2014). Given the complex geologic history, the presence of several isolated montane forest islands, and the wealth of diversity that has been discovered in nearby regions, it is clear that montane agamid lizards remain under studied and are an excellent group for better understanding the biogeographic history of the region (Harvey et al. 2014).

There has been considerable uncertainty regarding the taxonomic status of *Dendragama boulengeri*, which was considered to belong to a monotypic genus until only recently (Harvey et al. 2017). Early studies reported *D. boulengeri* from isolated montane forests of Jambi, west Sumatra (Sumatra Barat) and north Sumatra (Sumatra Utara) provinces (Doria 1874). However, Harvey et al. (2017) recently described two new species, *D. australis* from south Sumatra and *D. dioidema* from Aceh Province. They also provide a thorough redescription of *D. boulengeri*. In their paper, *D. boulengeri* is redescribed as a species distributed throughout much of Central Sumatra, including the type locality (Mount Singgalang in Sumatra Barat) and nearby Mount Marapi (paralectotype locality).

Previously, a population of *Dendragama* was described as *Acanthosaura schneideri* (Ahl 1926) from Sumatra Utara Province. The species was mentioned infrequently thereafter. In an unpublished dissertation, Moody (1980) conducted a thorough family-wide review of the Agamidae and split the genus *Calotes* into four genera: *Bronchocela*, *Calotes*, *Dendragama*, and *Pseudocalotes*. However, there is no mention of *A. schneideri* in his work. Only later did Manthey and Grossmann (1997) transfer *A. schneideri* to the synonymy of *D. boulengeri*. To date, there has been no phylogenetic analyses of *Dendragama* to confirm the status of the population from Sumatra Utara in relation to other populations.

We examined a series of *Dendragama* collected by MBH and ENS from their herpetofaunal inventory conducted between 2012 and 2014 throughout the Barisan Mountain Range of Sumatra. These samples include specimens from various isolated mountain peaks across much of central, northern, and southern Sumatra. Using an integrative analysis, we investigate species boundaries among these populations and biogeographic patterns revealed by the phylogenetic analyses.

## Materials and methods

### Biological inventory

A thorough herpetofaunal survey was conducted across the highland forests of Sumatra’s Barisan Mountain Range between 2012 and 2014. An international team of collaborators systematically targeted mountains based on geographic isolation from one another. We predominantly collected specimens at night. In total, we collected 195 specimens of *Dendragama* from localities throughout the highlands of Sumatra between 1231–2253 m elevation. We recorded GPS coordinates and ecological data on site. Animals were euthanized following appropriate IACUC protocols and DNA samples were taken for future identification prior to preservation in 10% formalin. Photographs were taken before and after euthanizing.

### Counts and measurements

**Figure 1.**
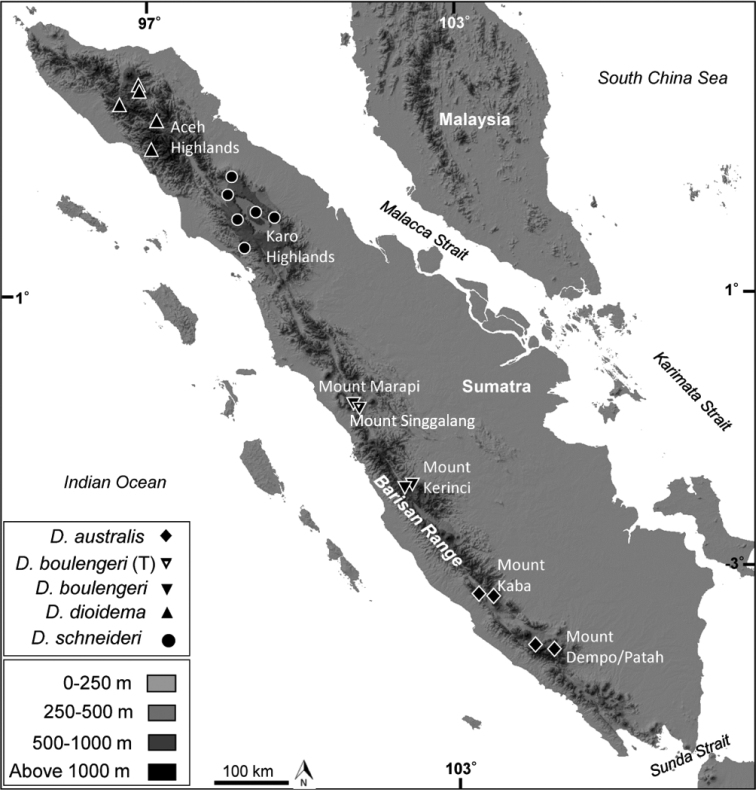
Collection localities of specimens of *Dendragama* used for comparisons in this study. (T) designates type locality and nearby paralectotype locality (Marapi). White dots represent hypothesized potential locations for new species or divergent populations. Dotted white lines show major biogeographic breaks.

We scored 32 different morphological characters for each specimen from the three populations of *D. boulengeri*. To avoid systematic errors introduced by separate observers, K. Shaney collected all mensural and meristic characters. Sex was determined by examining the gonads. We examined 19 *D. boulengeri* collected from Mount Marapi, west Sumatra (Marapi population), nine specimens from Mount Kerinci and Mount Tujuh, Jambi (Kerinci population), and 15 specimens from various mountains across the Karo Highlands of Sumatra Utara, collected near the type locality of *A. schneideri* (Karo population; Fig. 1).

Because measurements and scores are often done differently depending upon the study, some of the morphological characters used here require further comment (Harvey et al. 2014). We measured eye–nostril distance from the anterior ocular angle (bony edge of the orbit) to the tip of the snout. We consider the last supralabial to be lower and more elongate than the supralabials in front of it, and the last infralabial is positioned directly below the center of the last supralabial. We counted gular scales beginning immediately behind the mental (or the first pair of infralabials when in medial contact) until the transverse crease where the gulars meet the pectoral region. From the crease, we counted ventral scales to the last scale on the anterior edge of the cloacal opening. We counted nasal–rostral scales as the number of scales between the nasal and rostral. Postrostral scales were counted as the number of scales contacting the rostral which were not supralabials. We counted canthals as the number of scales between the nasal scale and the first supraocular scale. The number of scales between the supralabials and the first canthal touching the nasal scale are described here as the “scales between first canthal and supralabial”. Circumorbital scales include a canthal and the postciliary modified scale. We counted postmentals (or chin shields) if they contacted the infralabials. The number of midbody scales around the body included the ventrals and a scale of the dorsal crest. We counted lamellae on the fourth digit of the hands and feet, starting from the interdigital skin at the base of the digit and extending to the claw (i.e., including the elongate ungual scale). We counted nuchal crest scales from the first projecting scale to the last enlarged scale before the pectoral gap. Only projecting scales were counted and small flat vertebrals and paravertebrals that interrupt the crest were excluded.

To the nearest 0.1 mm with digital calipers, we measured snout-vent length (SVL) from the tip of the snout to the anterior lip of the vent and tail length by straightening the tail along the edge of a ruler and measuring from the posterior edge of the vent to the tip of the tail. We measured head length from skin covering the posterior edge of the mandible to the tip of the snout, trunk length as the distance from the axilla to the groin, and hand and foot length from the proximal margin of the palm or sole to the tip of the claw on the fourth digit. We measured the brachium as the length of the entire humerus and the antibrachium from skin covering the proximal end of the ulna (antebracheal fold) to the base of the palm. We measured the shank as the length of the tibia. The proximal and distal ends were determined with the elbow or leg flexed 90°. We measured internarial distance as the distance between the upper edge of each nostril, bony orbit, and tympanum width from the anterior to the posterior edge of each. Additional specimens examined from sister taxa and outgroups are provided in Suppl. material 1.

### Statistical analyses

Using our mensural and meristic data we compared the Karo, Kerinci, and Marapi populations of *Dendragama boulengeri*. For meristic characters, we compared means between the three populations using Tukey’s test after confirming assumptions of normality (using the Shapiro-Wilk test) and homoscedasticity (using Levene’s test). *Dendragama boulengeri* populations from the Karo population represent the group that we hypothesize to be a distinct species, *D. schneideri*; however, we will continue to refer to this as the “Karo population” until the results section. Thus, for clarity between the text, figures, and tables, when we refer to *D. boulengeri* (Karo population) or *D. schneideri* we are referring to the same population.

When making comparisons among populations, we analyzed males only for head width and head length, because we found these traits to be sexually dimorphic in a preliminary study of our series from Marapi. To investigate sexual dimorphism and to compare mensural characters among populations, we used analysis of covariance treating SVL as a covariate. To avoid inflation of the type I error rate in our morphometric comparisons, we performed three additional calculations. First, we made Bonferroni corrections to the probability scores for the tests among populations (Glover and Mitchell 2016). Second, having identified several apparent morphometric differences in among populations, we then verified the difference by rerunning the analysis using a different measurement as covariate in each apparently different trait: eye-nostril distance as a covariate for comparisons among thigh lengths, length of brachium for comparisons among hand length and length of shank for comparisons among foot length. Third, as a final validation of these results, M. B. Harvey measured SVL and tail length of a separate sample of nine *Dendragama* housed in the MZB and compared them to his own measurements for specimens from the type locality and Marapi.

### DNA extraction and amplification

We digested tissue in 100 μL of lysis buffer, then added 5 μL of proteinase K (20 mg/ml) and incubated at 55 °C for 1–6 hours. After incubation, we added 1.8 μL of serapure beads (Rohland and Reich 2012) for every 1 μL of digested sample. DNA extraction was carried out following the same methods that are used in PCR cleaning protocols described in AMPure magnetic beads literature (Agencourt, Bioscience, Beverly, MA, USA).

Phylogenetic Analyses – We extracted genomic DNA from 17 specimens of Sumatran *Dendragama*, including: *D. boulengeri* from Mount Marapi (Marapi population), *D. boulengeri* from Mount Kerinci (Kerinci population), *D. boulengeri* representing the Karo Highlands population (Mount Sibuatan, Mount Pangururan and Vicinity of Tele), *D. dioidema* (Mount Kaba, Mount Patah and Mount Dempo) and *D. australis* (Berni Terlong and Takengan). It’s important to note that individuals from the Karo Highlands were not collected precisely from the type locality of *A. schneideri* but were found in nearby geographic locations. We then combined new sequences from these specimens with sequences already published by Shaney et al. (2016) and Harvey et al. (2014, 2017) on GenBank. The published sequences include the outgroup taxa *Bronchocela cristatella* (Kuhl, 1820), *Lophocalotes ludekingi* (Bleeker, 1860), and *Pseudocalotes tympanistriga* (Gray 1831) see Suppl. material 2.

ND4 provided sufficient data for us to generate a phylogenetic hypothesis of *Dendragama*. We sequenced a fragment of the NADH dehydrogenase subunit 4 (ND4) gene using the forward primer “ND4” (CACCTATGACTACCAAAAGCTCATGTAGAAGC) and reverse primer “LEU” (CATTACTTTTACTTGGATTTGCACCA). The ND4 thermal cycle profile consisted of an initial denaturation at 94 °C for three minutes, followed by 30 cycles of denaturation at 94 °C for 30 seconds, a 50 °C annealing phase for 45 seconds and a 72 °C extension for one minute, followed by a 72 °C extension for seven minutes, then a holding phase at 4 °C.

All sequences were aligned using the Geneious aligner implemented within Geneious v. 6.1.8 (Kearse et al. 2012). ND4 sequences range in length from 616 to 934 bp. We identified the most likely model of evolution for each codon position using Bayesian information criteria implemented in PartitionFinder (Lanfear et al. 2012). We partitioned codon positions using GTR+ Γ. We conducted maximum likelihood analyses using raxmlGUI (Sylvestro et al. 2012). We utilized the thorough bootstrapping setting, sampling over 10 runs of 10,000 repetitions. We carried out Bayesian phylogenetic analysis using MrBayes v3.2.1 (Ronquist and Huelsenbeck 2003). We used four independent runs (nruns = 4) and four chains (three heated chains and one cold chain) for 10 million generations, sampling every 100 generations. We discarded the first 25% of samples as burn-in (Rambaut et al. 2014). We confirmed adequate mixing and assessed the appropriate amount of burn-in and convergence by inspecting the trace files in the program TRACER v1.6 (Rambaut et al. 2014). We conducted UPGMA analyses and calculated uncorrected pairwise distances using Mega 5.1 (Tamura et al. 2011).

## Results

### Phylogenetics and biogeography

**Figure 2.**
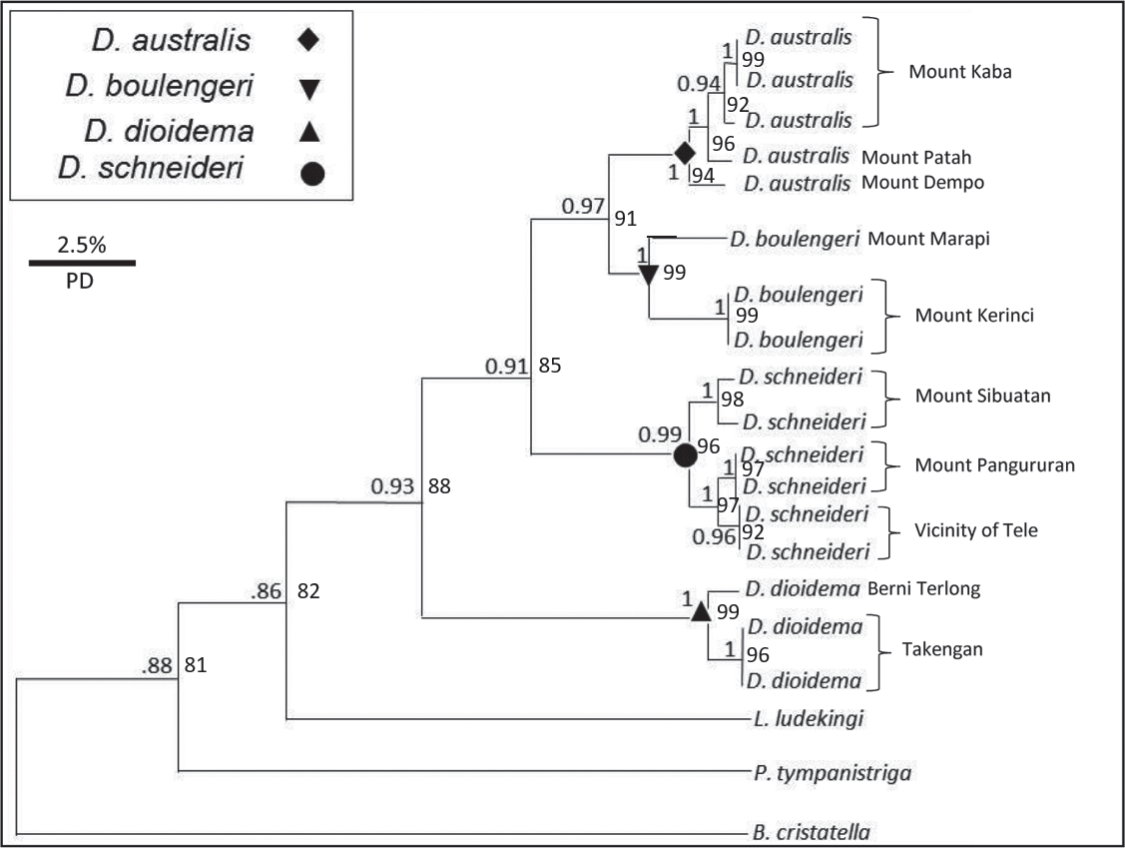
Bayesian tree of *Dendragama* and other agamid taxa included in our analyses. PD = Pairwise Distance. Mountains associated with *Dendragama* population sampling are noted. *Bronchocela* abbreviated as B, *Lophocalotes* as L, and *Pseudocalotes* as P.

Both our Maximum Likelihood and Bayesian Analyses revealed the same relationships within *Dendragama*, the only difference being that the ML tree returned slightly lower support values (Fig. 2). Both analyses found *Lophocalotes* to be sister to *Dendragama*, followed by insular *Pseudocalotes*. We refer to *Pseudocalotes* from Java and Sumatra as “insular” because they are not closely related to *Pseudocalotes* from mainland Asia (Harvey et al. 2014).

Within *Dendragama* five clades have strong nodal support (posterior probabilities between 0.98 and 1.00): *D. australis* (Mount Dempo, Kaba and Patah region), *D. boulengeri* (Mount Kerinci), *D. boulengeri* (Mount Marapi), *D. dioidema* (Aceh Highlands) and *D. boulengeri* (Karo Highlands). For clarity, the Karo Highlands populations will be discussed in the redescription section of *D. schneideri*. Each of these clades is restricted to geographically isolated locations. Dispersal between populations is highly unlikely to occur given that all *Dendragama* rely on cloud forest habitat which is not continuous between any of these populations.

*Dendragama schneideri* from the Karo highlands represent a genetically distinct group from central Sumatra and includes all specimens from Mount Sibuatan, Mount Pangururan and the Vicinity of Tele (Fig. 2). *Dendragama boulengeri* populations from Mount Marapi (paralectotype locality of *D. boulengeri*) and *D. boulengeri* populations from Mount Kerinci are geographically isolated by elevation and breaks in montane cloud forest patches. Based on biogeographic patterns, there are 18 distinct localities that might hold new species of *Dendragama*.

### Genetic distances

The ND4 gene is 5.0–12.1% distant (Table 1) among *D. australis*, *D. dioidema* and the three populations of *Dendragama boulengeri*. At the lower extreme, 5.0% distance separates the Marapi and Kerinci populations of *D. boulengeri*. While *D. boulengeri* from the Karo population are 6.0–8.0% divergent from Marapi and Kerinci populations.

**Table 1.** Uncorrected pairwise genetic distances (ranges) for ND4 sequences between populations of *Dendragama* (including 5 species described by Harvey et al. (date), *Lophocalotes ludekingi* and *Pseudocalotes tympanistriga*. For *Dendragama*, M = Marapi population, K = Kerinci population, Ka = Karo population.

Species	*D. boulengeri* (M)	*D. boulengeri* (K)	*D. schneideri* (Ka)	*D. australis*	*D. dioidema*	*L. ludekingi*
*D. boulengeri* (K)	5%					
*D. schneideri* (Ka)	6.0–7.0%	7.0–8.0%				
*D. australis*	6.0–6.4%	6.0–6.4%	7.0–8.0%			
*D. dioidema*	10.7–12.1%	9.0–10.1%	9.0–10.1%	10.7–11.7%		
*L. ludekingi*	16–17%	16–17%	16–17%	16–17%	16–17%	
*P. tympanistriga*	19–20%	19–20%	19–20%	19–20%	19–20%	19–20%

*Dendragama dioidema* is the most distant from species. Its ND4 gene is 10.7–11.7% distant from *D. australis*, and 10.7–12.1% from populations of *D. boulengeri*. In contrast, ND4 sequences of *D. australis* (south Sumatra) are 6.0–6.4% distant from populations of *D. boulengeri.*

The Karo population of *Dendragama boulengeri* (Now *D. schneideri*) has diverged by 6.0–8.0% from the Marapi and Kerinci populations and by 6.0–11.9% from *D. australis* and *D. dioidema*.

### Morphology

**Figure 3.**
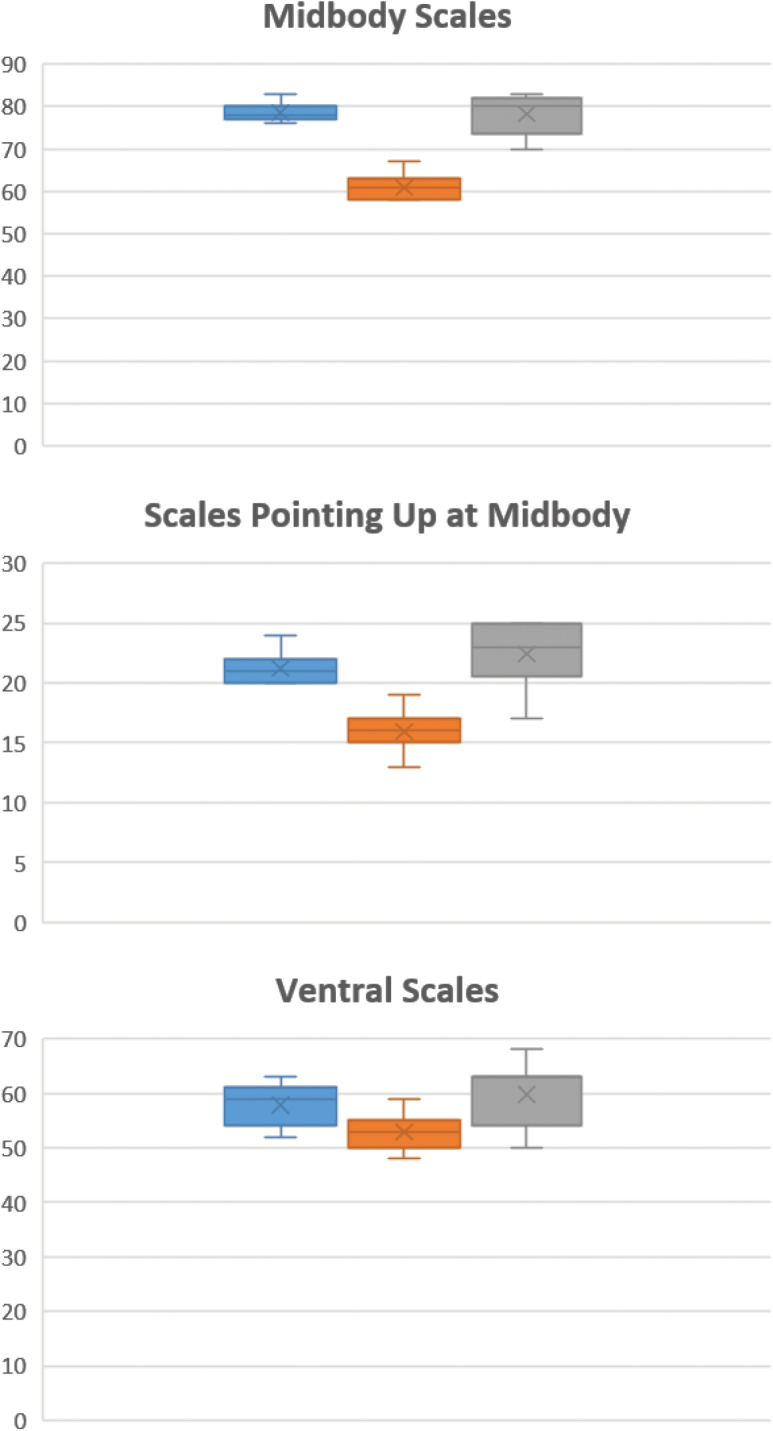
Box-and-whisker plots of three statistically significant different characters compared: **A** midbody scale counts, **B** scales pointing up at midbody, and **C** ventral scales. Scales abundance is provided on the Y axis and the three species are provided in different colors: blue for *D. boulengeri* (Marapi), orange for *D. schneideri*, and grey for *D. boulengeri* (Kerinci).

A suite of meristic characters distinguishes the Karo population of *D. boulengeri* from the other two populations. Specimens from Karo have statistically significant differences in several characters (Tukey Test), including fewer scales around midbody, fewer ventral scales, and large heterogeneous scales along the flanks (Table 2). The Marapi population of *D. boulengeri* has more scales between the nuchal and dorsal crest and subdigital lamellae than the other two populations. Finally, *D. boulengeri* specimens from Kerinci have fewer circumorbitals (11–13) than specimens from Marapi and Karo (usually 15) (Tables 2 and 3). We did not find interpopulation differences for the other meristic characters (P > 0.05). Fig. 3 provides a visual of some of the relationships among characters.

**Table 2.** Measurements of *D. schneideri* and *D. boulengeri* populations. Ranges are followed by average ± standard deviation in parentheses.

Measurement	*D. boulengeri* (Marapi population, n = 19)	*D. boulengeri* (Kerinci population, n = 9)	*D. schneideri* (Karo population, n = 15)
Flank/Pectoral Width	2.58–4.21% (3.54 ± 0.43)	2.57–4.99% (3.20 ± 0.92)	2.41–3.41% (3.03 ± 0.32)
Thigh/Shank Length	1.02–1.55% (1.31 ± 0.15)	1.46–1.67% (1.55 ± 0.08)	1.26–1.70% (1.53 ± 0.12)
Brachium/Anti. Length	0.90–1.29% (1.11 ± 0.08)	0.96–1.40% (1.13 ± 0.14)	0.93–1.21% (1.10 ± 0.08)
Snout Vent/Tail Length	1.96–2.43% (2.22 ± 0.43)	2.02–2.23% (2.11 ± 0.92)	2.02–2.46% (2.08 ± 0.32)
Head Length/Head Width	1.47–1.77% (2.0 ± 0.29)	1.29–1.66% (1.48 ± 0.14)	1.26–1.83% (1.62 ± 0.18)
Max. Snout–Vent Length	78.13 mm	80.56 mm	79.2 mm
Nasal to Rostral Scales	1–2, 1 (95%), 2 (5%)	1 (100%)	1 (100%)
Nasal to Sup. Scales	0–2, 0 (58%), 1 (37%), 2 (5%)	0 (100%)	0 (75%), 1 (25%)
Post Rostral Scales	5 (100%)	5 (100%)	5–6, 5 (91%), 6 (9%)
Canthals (Nasal to Sup.)	5–7, 5 (74%), 6 (21%), 5%)	5–6, 5 (83%), (17%)	5–7, 5 (45%), 6 (45%), 7 (9%)
Loreal Scales	5–6, 5 (89%), 6 (11%)	6–7, 6 (50%), 7 (50%)	6–7, 6 (73%), 7 (27%)
Scales Canth. and Sup.	2–4, 2 (5%), 3 (90%), 4 (5%)	2–3, 2 (17%), 3 (83%)	2–3, 2(37%), 9 (63%)
Circumorbital Scales	13–16, 13 (37%), 14 (53%), 15 (5%), 16 (5%)	11–13, 11 (17%), 12 (66%), 13 (17%)	13–15, 13 (73%), 14 (18%), 15 (9%)
Scales Nuch. and Dor.	8–10, 8 (47%), 9 (21%), 10 (26%), 11 (5%)	6–9, 6 (17%), 7 (33%), 8 (33%), 9 (17%)	5–9, 5 (9%), 6 (9%), 7 (36%), 8 (18%), 9 (27%)
Scales up at Midbody	20–24 (21.21 ± 1.27)	20–25 (23.66 ± 1.9)	13–19 (16 ± 1.95)
Midbody Scales	77–84 (79.57 ± 1.89)	75–89 (84.16 ± 4.99)	59–68 (62.36 ± 2.8)
Gular Scales	35–43 (38.95 ± 2.01)	34–42 (37.89 ± 2.97)	32–44 (36.81 ± 3.51)
Ventral Scales	52–63 (57.89 ± 3.71)	56–68 (62.16 ± 3.97)	48–59 (52.45 ± 3.14)
Sub. Lamellae of Toe IV	27–36 (30.42 ± 2.38)	25–31 (28.5 ± 2.58)	25–32 (28.09 ± 2.02)
Sub. Lamellae of Finger IV	24–31 (27.42 ± 1.95)	22–24 (23.16 ± 0.75)	22–26 (24.27 ± 1.19)
Supralabials	9–10, 9 (58%), 10 (42%)	8–10, 8 (50%), 9 (33%), 10 (17%)	9–10, 9 (91%), 10 (9%)
Infralabials	8–11, 8 (21%), 9 (53%), 10 (21%), 11 (5%)	8–9, 8 (67%), 9 (33%)	8–9, 8 (45%), 9 (55%)

**Table 3.** Results of Tukey’s Tests. The three *Dendragama boulengeri* populations (Karo Highlands, Kerinci, and Marapi) were compared and statistically significant results show that the Karo Highlands population is morphologically distinct from Kerinci and Marapi populations. The Karo Highlands population should be referred to as *D. schneideri*.

**Character**	**Tukey’s *Q*, probability**	
	**Marapi**	**Kerinci**
Circumorbitals		
Karo	NS	5.00, 0.003
Kerinci	8.21, 0.000	
Scales between nuchal and dorsal		
Karo	4.16, 0.015	NS
Kerinci	4.92, 0.004	
Dorsals pointing upward		
Karo	10.89, 0.000	13.44, 0.000
Kerinci	NS	
Scales around midbody		
Karo	21.3, 0.000	20.8, 0.000
Kerinci	NS	
Ventral scales		
Karo	4.40, 0.010	6.04, 0.000
Kerinci	NS	
Lamellae under toe 4		
Karo	3.89, 0.024	NS
Kerinci	3.82, 0.027	
Lamellae under finger 4		
Karo	7.81, 0.000	NS
Kerinci	10.2, 0.000	
**Character**	**ANCOVA *F*, Bonferroni corrected probability**	
	**Marapi**	**Kerinci**
Tail length		
Karo	NS	15.57, 0.002
Kerinci	6.77, 0.046	
Hand length		
Karo	NS	11.25, 0.009
Kerinci	11.61, 0.007	
Foot length		
Karo	NS	10.51, 0.012
Kerinci	17.8, 0.001	
Orbit		
Karo	Nonparallel (18.94, *P* < 0.001)	NS
Kerinci	8.67, 0.021	
Thigh length		
Karo	NS	7.91, 0.031
Kerinci	NS	

Male *Dendragama boulengeri* from Marapi have wider (*F_1,16_* = 9.08, *P* = 0.008) heads than females and width of their heads increases faster during ontogeny (*F*_equal slopes_ = 6.50, *P* = 0.022). Although just not significant if 0.05 is chosen as the type I error rate, male *D. boulengeri* from Marapi also have longer heads (*P* = 0.072) than females. With small samples sizes from Karo and Kerinci, we lacked sufficient statistical power to confirm sexual dimorphism in head size (*P* > 0.2). Nonetheless, males from Karo appear to follow the same growth trajectory. We could not demonstrate sexual dimorphism in our meristic characters or in tail length, eye–nostril length, pectoral width, or length of the body (*P* > 0.26).

*Dendrama boulengeri* specimens from Kerinci have relatively shorter tails, hands, and feet than specimens from the other two *D. boulengeri* populations. They also have shorter thighs than specimens from Karo and a smaller orbit than specimens from Marapi. Small specimens from Karo have a relatively smaller orbit than small specimens from Marapi; however, orbits are about the same size for larger specimens from the two populations. Our limited data suggests a different growth trajectory for the orbit at Karo vis-à-vis Marapi, but having violated the assumption of parallel regression lines, we do not report a probability for this comparison between Karo and Marapi. As detailed in the methods, we confirmed each of these morphometric differences by treating other measurements as covariates. Moreover, a separate sample of nine *Dendragama* from Kerinci had relatively shorter tails (*F*_1,27_ = 7.75, *P* = 0.010) than the sample of *D. boulengeri* from both Marapi and the type locality described by Harvey et al. (2017). We did not find differences among populations for eye–nostril distance, pectoral width, length of body, length of shank, length of brachium, length of antebrachium, or internarial distance (P > 0.12). Tables 2 and 3 provide statistics for the three populations compared.

In addition, *Dendragama* from the Marapi and Kerinci populations have a bright yellow buccal epithelium and tongue, whereas lizards from the Karo population have a pink to red buccal epithelium and tongue. Along their lower flanks, lizards from Karo have numerous distinctly enlarged tubercular scales. In contrast, specimens from the other populations lack these scales.

### Species delineation

**Figure 4.**
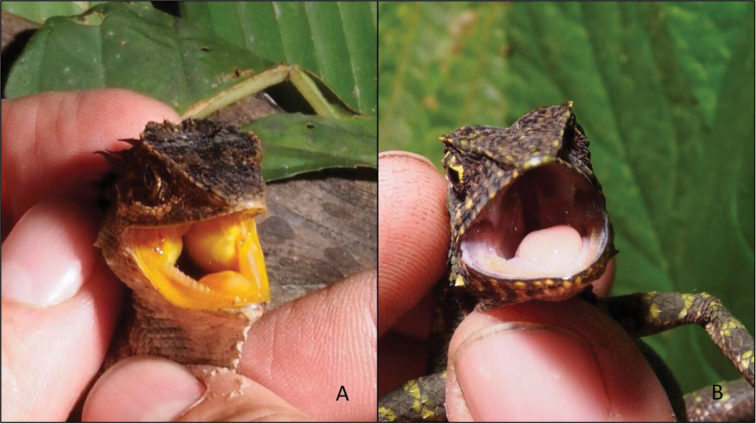
The bright orange mouth of *Dendragama boulengeri* is shown in **A**, whereas the pink mouth of *D. schneideri* is shown in **B** (photographs by ENS).

Our analysis revealed numerous differences between the Karo population on the one hand and the Marapi and Kerinci populations on the other. Numerous different means and high genetic divergence is evidence of an interruption in gene flow among these populations, but is of only limited diagnostic value. However, we also identified four fixed characters that distinguish the Karo population from the other two. Unlike these populations (characters in parentheses), the Karo specimens have pink to red buccal epithelia (yellow, Fig. 4), numerous enlarged tubercles on the lower flank (scales of lower flanks homogenous or with few slightly enlarged scales), 59–68 scales around midbody (77–89), and 13–19 dorsals pointing upward and backward at midbody (20–25). Restricted to highland areas above 1,200 m, inhospitable lowlands isolate the Karo population from all other populations and species of *Dendragama*. Direct comparison of Ahl’ (1926) type of *Acanthosaura schneideri* to the Karo specimens reveals that they are the same species. Accordingly, our analyses support the removal of *A. schneideri* Ahl, 1926 from the synonymy of *Dendragama boulengeri* Doria, 1888. Hereafter the Karo population should be recognized as a distinct species *Dendragama schneideri*.

#### Redescription of Dendragama schneideri

Taxon classificationAnimaliaSquamataAgamidae

472F0E5D-822D-58E5-86B1-9705FD60F456

Acanthosaura schneideri Ahl 1926: 186, Simbolon, Battaker-Hochebene, Sumatra.
Dendragama boulengeri : Werner 1900, Schenkel 1901, De Rooij 1915 (in part): 119, Wermuth 1967, Häupl 1994, Manthey and Grossmen 1997 (in part): 166–167, Denzer et al. 1997, Hallermann 2000.

##### Holotype.

**Figure 5.**
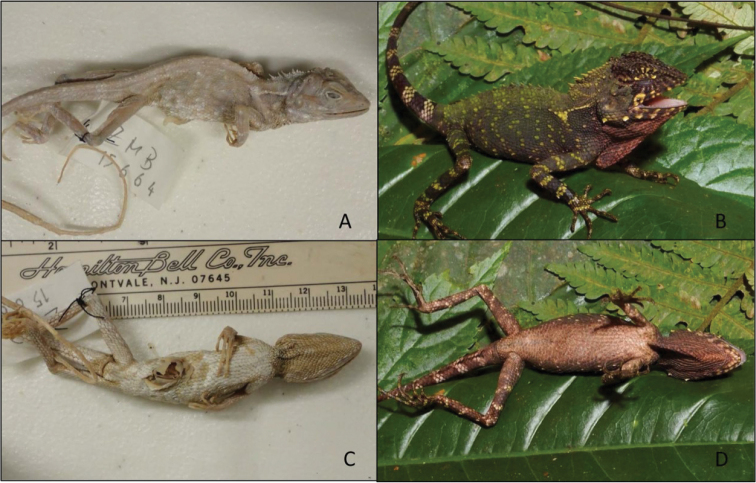
**A, C** holotype of *D. schneideri* from two different angles (photographs by MBH) **D** specimen MZB 12098 from the same angles (photographs by ENS).

An adult male (ZMB 15664, Fig. 5) from “Simbolon, Battaker-Hochebene, Sumatra (= Simbolon, Sumatra Utara Province, Indonesia.)”. Approximate GPS locality 3.005538°N, 98.923650°E.

##### Referred material.

All specimens were collected in Sumatra Utara near the type locality. Four specimens (UTA 62872, 2.91032°N, 98.4516°E; UTA 62873, 2.91329°N, 98.46091°E; UTA 62874, 2.9121°N, 98.46222°E; MZB 14126, 2.91189°N, 98.46538°E) from Mount Sibuatan, 1595–1883 m. Two specimens (UTA 62863, 3.2143°N, 98.49955°E; MZB 14127, 3.2143°N, 98.49955°E) from Sibayak, 1550 m. Two specimens (UTA 62865, 3.22576°N, 98.51974°E; UTA 62866, 3.20637°N, 98.51974°E) from the vicinity of Peceran, 1530–1727 m. One specimen (UTA 62870, 2.5911°N, 99.93921°E) from Mount Pangulubao, 1258 m. One specimen (UTA 62871, 2.1706°N, 98.63612°E) from an unnamed road near Onan Ganjang, 1231 m. One specimen (MZB 12098, 2.56103°N, 98.59106°E) from the vicinity of Tele, 1768 m.

##### Diagnosis.

**Figure 6.**
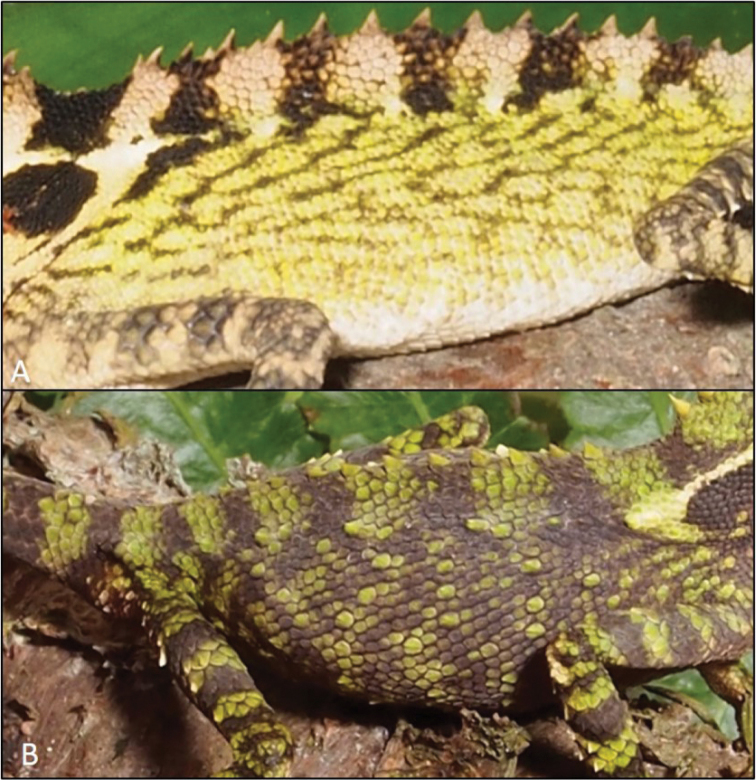
**A** flank of male *Dendragama boulengeri* (MZB 9825) and its thin, horizontal banding patterns, thick vertical bands along dorsal crest, small homogenous scales, and lack of enlarged, keeled scales **B** flank of male *D. schneideri* (UTA 62868) and its lack of horizontal banding along the flanks, thin vertical bands along dorsal crest, large heterogeneous scales, and enlarged, strongly keeled scales dispersed across the flanks (photographs by ENS).

A species reaching at least 201 mm in total length (SVL) and distinguished from congeners by the following characters: (1) midbody scales 58–67; (2) dorsal scales heterogeneous across flanks (Fig. 6); (3) strongly keeled white/yellow scales randomly distributed along flanks (more numerous and distinct in females); (4) ventral scales 48–59; (5) banding pattern along flanks often muddled, but typically vertical when present; (6) mouth and tongue pink to red in life; (7) narrow, vertical black stripes across dorsal crest, limbs, digits and most of tail; (8) female color in life dark brown, yellow and black with amber coloration on underside, while males green and lacking amber coloration along ventral surface; (9) dorsal and nuchal crest clearly separated by 5–9 dorsal scales; (10) dorsal crest serrate, extending to base of the tail, comprised of 23–31 projecting, triangular scales; (11) a series of 3–4 enlarged tubercles present along the chin of males and females; (12) A series of 12–18 strongly keeled, white/yellow femoral spines present (combined count on both sides).

##### Description and variation.

The description is based on the 19 referred specimens. Where appropriate we provide character state frequencies or means ± standard deviation in parentheses. When available and not subject to interobserver biases, we also provide data gathered by MBH for the holotype in brackets.

Flank/pectoral width 2.41–3.41 (3.03 ± 0.32); thigh/shank length 1.26–1.70 (1.53 ± 0.12); brachium/antibrachium length 0.93–1.21 (1.10 ± 0.08); SVL/tail length 2.02–2.46 (2.24 ± 0.11); head length/head width 1.26–1.83 (1.62 ± 0.18); snout–vent length 61.35–79.2 mm (68.82 ± 5.59) (74 mm, tail length 145 mm).

Supralabials smooth, nine (91%) or 10 (9%); infralabials smooth eight (45%) or nine (55%); supraocular scales five (82%) or six (18%); postrostrals small, five (91%) or six (9%) [5]; scales between nasal and rostral one (100%); nasal separated from supralabials by small lorilabials (75%) or contacting first supralabial (25%); canthals from nasal to supraocular five (45%), six (45%), or seven (9%) [5]; loreal scales six (73%) or seven (27%), scales between first canthal and supralabials two (37%) or three (63%); circumorbitals 13–15, usually 11 (73%); postmentals contacting infralabials one (9%) or two (91%); first pair of postmentals in medial contact (66%) or separated by one gular (34%) [1].

Nuchal crest clearly separated from dorsal crest and gap between crests spanning 5–9 scales; dorsal crest serrate, continuous down to tail; scales on dorsum, large and heterogeneous, with series of enlarged strongly keeled, yellow/white scales in row below dorsal crest; all other scales along dorsum and flank smooth to feebly keeled; scales along flank consistent with dorsum, with more enlarged strongly keeled scales in vertical rows along sides; midbody scales 58–67 (61.36 ± 2.8) [61], gulars smooth 32–44 (36.81 ± 3.51) [30]; ventral scales 48–59 (52.45 ± 3.14) [52], ventrals keeled from chest to lower abdomen before transitioning to smooth scales near precloacal area; precloacal scale width small 0.75–1.4 (1.02 ± 0.22); scales along limbs strongly keeled, with continuation of keeled scales down to fingers on both hands and feet; subdigital lamellae on finger IV 22–26 (24.27 ± 1.19) [23]; subdigital lamellae on toe IV 25–32 (28.09 ± 2.02) [27]; dorsal crest scales 23–31 (26.63 ± 2.69) [28].

##### Coloration in life.

There is distinct sexual dichromatism in this species and coloration changes in all *Dendragama* in response to rough handling. Females of *Dendragama schneideri* are typically shades of dark brown, green, and black with vertical black and yellow bands running along the extent of the dorsal crest. Bands extend almost to the end of the tail; tail bands 14–18 (12.2 ± 0.83), and enlarged green, yellow or white, strongly keeled scales are present intermittently along the flanks. Black and yellow/green bands also extend along all limbs, hands, and feet. A black spot is present under the base of the nuchal crest as in other species of *Dendragama*. The throat has amber and brown coloration, which may or may not be broken up by small lateral brown lines. Brown and amber coloration extends along the lower flanks and all the way to the end of the tail. Yellow and black lines radiate around the eyes and across much of the face. Yellow, green or white enlarged tubercles are present below the eye and ear, and the mouth is pink to red.

**Males** may also be brown but are typically much lighter in coloration. They are often bright green and yellow with incomplete stripes of black scales, which zigzag vertically along the flanks. Black bands extend along the length of the dorsal crest and throughout the extent of the tail. Bands also cross the arms, legs, hands, and feet. A black prescapular blotch is present under the base of the nuchal crest, but may be less pronounced in some specimens. The venter is much lighter than in females, with a white or cream gular region, with some brown shading along the ventral side. Darker individuals may have some brown shading along the gular region as well. Green or yellow and black stripes radiate out from the eyes and, as with females, the mouth is pink to red.

##### Etymology.

The name “*schneideri*” honors Gustav Schneider (17 January 1867–14 April 1948).

**Standard English name.** Schneider’s Tree Agamid

##### Distribution and natural history.

**Figure 7.**
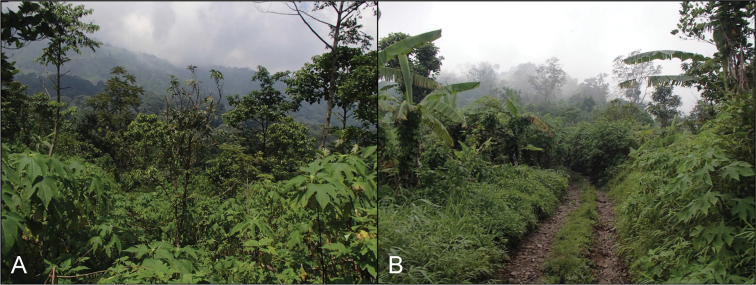
**A, B** highland cloud forest habitat of *Dendragama schneideri* (Photographs by ENS). Photographs taken at Mount Kerinci, Sumatra Barat Province illustrating similar habitats to cloud forests of the Karo Highlands where *D. schneideri* is found.

*Dendragama schneideri* occurs in high elevation, montane forest in north Sumatra’s Bukit Barisan Mountain Range (Figs 1, 7). Our sampling encompasses Lake Toba (where the Toba blast occurred 71.6 kya) and the surrounding Karo Highlands. We hypothesize that the northern latitudinal limit of *D. schneideri* occurs at a break in the Barisan Range where elevation drops down into valley floors below 1000 m and does so continuously from the eastern to western edge of the range. That break in topography would certainly prohibit dispersal between montane sky islands today. Yet, maybe more importantly in terms of maintaining population differentiation, elevational ranges that drop below that point would have even prohibited dispersal during periods of glacial maxima during the Pleistocene epoch when montane forests retreated downslope 300–500 m (Hall 2009). The break occurs approximately at 3.196889°N 98.102583°E and we only found populations of *D. dioidema* north of that point. We hypothesize that the southern latitudinal limit of *D. schneideri* occurs approximately near the border of Sumatra Utara and Bengkulu. Similar to the northern limitation, there is a continuous elevational break that drops below 1000 m that extends across the width of the Barisan Range at the southern end of their range.

All the referred specimens were found sleeping in low vegetation 0.7–2.5 m above ground and between 1200–2800 m. All were found in montane cloud forest habitat and in higher elevations some individuals were found on moss covered vegetation in stunted forests where temperatures were slightly lower. Because the forests they inhabit receive high levels of precipitation relatively evenly distributed throughout cloud forests, *D. schneideri* are not dependent upon water bodies. Although some individuals were located along slopes near the edges of streams, those encounters did not seem to occur in any higher frequency than individuals being found in other areas away from bodies of water.

Many of the individuals collected were found along the edges of roadways and thus it seems they survive well along the edges of disturbed habitat, although they seem to be cloud forest obligates and require the presence of montane forest in some amount in order to persist in fragmented habitat.

Virtually nothing is known about the home range sizes and movement patterns of *D. schneideri*; however, given that they are relatively small arboreal lizards and live in cooler temperature habitats we would expect that they don’t move long distances throughout the year and do not have large home ranges.

We documented distinct sexual dichromatism. Because the males tend to be more brightly colored, we suspect they use their brightly colored dewlaps to display during mating season. It is also likely they use their displays to defend territories, as do nearly all other brightly colored lizards (Sinervo and Lively 1996; Steffen and Guyer 2014). They may also use their colors when competing with other individuals. Although little is known about the life cycles of *D. schneideri* specifically, Harvey et al. (2017) suggest that other species of *Dendragama* reach maturity at around 60 mm, females lay 2–4 ovoid eggs and probably produce multiple clutches each year. This may also be true for *D. schneideri* given their other similarities in life histories. Little is known about the diet of *D. schneideri*; however, they are suspected to be insectivores.

### Key to the species of *Dendragama*

**Figure 8.**
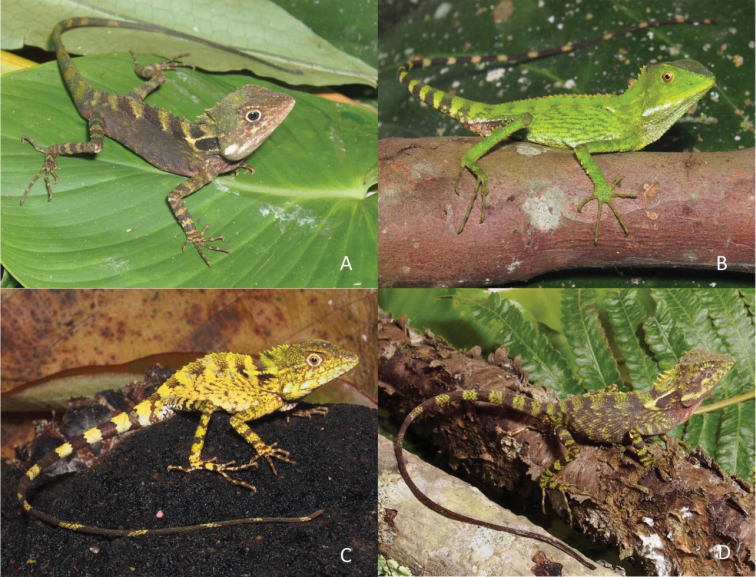
Male representatives currently recognized *Dendragama***A***D. boulengeri* ENS 19656 **B***D. australis* ENS 18556 **C***D. dioidema* ENS 19433 **D***D. schneideri* UTA 62868 (photographs by ENS).

We present a key to the species of *Dendragama* based on morphology and color pattern. Fig. 8 illustrates all four species. High supratemporal ridges enclosing a depressed parietal region, a row of white to yellow sublabial tubercles, and a visible tympanum immediately distinguish species of *Dendragama* from all other Sumatran agamids.

**Table:** 

1	Gular scales *large*, 15–30, ventrals smooth to feebly keeled	**2**
–	Gular scales *small*, 32–42	**3**
2	*Small* midbody scales, 61–94, mouth and tongue orange to yellow; short white sublabial stripe extending from below the eye to below (or just behind) the ear; brown band on neck, no large black prescapular blotch absent; proximal half of tail with 8–13 dark brown or green bands	***D. australis***
–	Midbody scales *moderate* in size, 57–77; mouth and tongue pink to red; no distinctive prescapular blotch present; proximal half of tail with 6–10 dark brown or green bands; *no* white sublabial stripe, however one or two white or pale yellow spots *present*	***D. dioidema***
3	*Large* midbody scales, 59–68, ventrals strongly keeled, upper dorsals 13–19 directed upward and backward; mouth and tongue pink to red; enlarged tubercles present on lower flanks	***D. schneideri***
–	*Small* midbody, 74–88, ventrals heavily keeled, upper 20–25 dorsals directed upward and backward; mouth and tongue yellow; few weakly keeled scales along lower flanks, enlarged tubercles absent	***D. boulengeri***

## Discussion

Morphological and molecular data presented here show the clear distinction between *D. boulengeri* and *D. schneideri*. *Dendragama schneideri* is geographically isolated from other species and only distributed throughout the Karo Highlands. It occurs in high elevation cloud forest allopatric from *D. australis*, *D. boulengeri*, and *D. dioidema*. Based on the lack of biological inventory in other parts of the Barisan Range, it is likely that other undescribed species of *Dendragama* may occur across the region.

### Biogeography

Using biogeographic patterns of genetic variation among *Dendragama* we identified at least 18 mountains that are likely candidates for new species. Moving from north to south, we estimate the break between the *D. schneideri* and *D. dioidema* lineages probably occurs where low elevation valleys run between Mount Sinabung and Mount Sibuatan slightly to the west of the Aceh-Sumatra Utara provincial border. For reference, the point 3.196889°N 98.102583°E is approximately along the line where this break occurs. We suggest that area as a likely break because that seems to be where topography of the Barisan Range drops to its lowest point between the distributions of those groups. Among *D. dioidema* lineages there is distinct genetic variation between populations in the Leuser Mountains and the Boundahara Mountains, which are divided by a low valley running north and south, which is paralleled by the Blangkejeren-Kutacane Road.

Based on Shaney et al. (2020) breaks in species boundaries of *Dendragama* seem to occur consistently wherever elevation dips to 650–700 m (or lower) between mountains based on glacial periods during the Pleistocene. Thus, we hypothesize the following mountains will hold new *D. dioidema* sister lineages (Table 4).

**Table 4.** Estimated geographic limitations of species boundaries and hypothesized locations where new species of *Dendragama* are presumed to be found based on biogeographic patterns. PNS = potential location of new species, SB = species boundary. Locations ordered from northern to southern latitude.

Feature	Coordinates	Locality
PNS	5.445944, 95.662639	Cot seulawah Agam
PNS	5.042222, 95.634722	Gunung Hulumasen
PNS	5.371194, 95.348500	Aceh Besar Regency
PNS	4.811722, 96.828694	Mount Bur ni Geureudong
PNS	4.921167, 96.350250	The mountains around Gunung Peuet Sagoe
PNS	4.636124, 97.411502	East Aceh Regency
SB	3.196889, 98.102583	Break in *D. dioidema* and *D. schneideri*
SB	1.418139, 99.243389	The break between *D. schneideri* and *D. boulengeri*
PNS	0.995028, 99.379694	South Tapanuli Regency
PNS	0.968111, 99.651917	Padang Lawas Regency
PNS	0.743472, 100.232889	Rokan Hulu Regency
PNS	0.060503, 99.984076	Mount Talakmau
PNS	0.209722, 100.299139	Pasaman Regency,
PNS	-0.341389, 100.678278	Mount Sago
PNS	-0.399639, 100.334917	Mount Singgalong
PNS	-2.503333, 101.874083	Mount Masurai
PNS	-3.397250, 102.347028	Bukit Daun
SB	-3.489034, 102.535034	Break between *D. boulengeri* and *D. australis*
PNS	-3.510812, 102.625556	Mount Kaba
PNS	-3.618861, 102.913278	Empat Lawang Regency
PNS	-3.893361, 103.259111	Lahat Regency
SB	-4.460662, 103.430325	Southern Limit of *Dendragama*

Further south in latitude, the Karo highlands surrounding the Toba eruption site consist of topography that is continuously connected by higher elevation pieces of terrain and thus, there are likely few new sister species of *D. schneideri* throughout the Karo highlands between the latitudes 3.196889°N, 98.102583°E and 1.418139°N, 99.243389°E. However, there is distinct population subdivision among the populations that we sampled.

The break between populations of *D. schneideri* and *D. boulengeri* likely occurs around the point 1.418139N, 99.243389E which we hypothesize based on the dramatic drop-off in elevation that cuts through the entire width of the Barisan Range throughout the area. South of that point, the Barisan’s topography becomes more variable in terms of having lower elevation valleys between mountains in a larger number of locations. Thus, we hypothesize the following nine mountains will have new sister species of *D. boulengeri*, including the mountains of the south Sapanuli Regency, Padang Lawas Regency, Rokan Hulu Regency, Mount Talakmau, Muaro Sungai Lolo of Pasaman Regency, Mount Sago, Mount Masurai, Bukit Daun, and Air Duku area of Rejang Lebong Regency. Not all of these ranges had clear names on the topographic maps, but for reference we provide GPS coordinates to the center point of the ranges in (Table 4).

The southern limitation of *D. boulengeri* populations probably occurs near the town of Curup where the mountains drop down very low in elevation, again separating *D. boulengeri* populations (and likely sister species) from *D. australis* populations. Among *D. australis* we hypothesize the following three mountains will hold new closely related species: Mount Kaba, Unknown strip of mountain in Empat Lawang Regency, Mountains in Sukabumi of Lahat Regency. The southern limitation of *D. australis* is not precisely known although we did not collect *Dendragama* further south in Latitude than Gunung Patah at -4.460662, 103.430325. We did collect *Pseudocalotes cybelidermus* and *P. guttalineatus* extensively throughout mountains further south, including Gunung Pesagi and montane forest above Ngarip and *P. rhammanotus* from Danau Ranau in Lampung Province. The absence of *Dendragama* from locations further south suggests populations of *D. australis* probably do not occur much further south than Gunung Patah.

We hypothesize that each mountain probably has a new species, not just the possibility of one or two new species among all mountains mentioned. Based on Shaney et al. (2020) findings regarding the elevational shifts of Pleistocene cloud forests, it is likely that all of the 18 mountains mentioned remained isolated even during periods of glacial maxima. This latter point is crucial because Pleistocene connectivity is what would have facilitated gene flow historically and sympatric distributions contemporarily (which is not how they are distributed). Similar patterns have been seen in among peninsular Malaysia’s herpetofauna as well (Loredo et al. 2013; Grismer et al. 2017). It is unlikely that *Dendragama* were able to disperse among any of the isolated mountains for millions of years, allowing for a whole array of species to diverge. Using these biogeographic patterns to identify likely locations for new species of *Dendragama* is certainly interesting on its own; however, perhaps more importantly, those same locations are probably where the distributions of many obligate cloud forest groups with limited dispersal (e.g. insects, rodents, amphibians) change in Sumatra. Thus, those mountains would be excellent candidates for other taxonomists conducting biological inventory throughout the region as well.

Additionally, a comparative biogeographic analysis among *Dendragama* and other agamid lizard groups would be a fascinating and informative study to better understand Sumatra’s complex biological history. Grismer et al. (2016) provided a biogeographic study of draconid lizards of southeast Asia that yielded fascinating information; however, species of draconids from Sumatra remain underrepresented in biogeographic analyses (Inger and Voris 2001; Cannon et al. 2009). Furthermore, genetic splits between agamid lizard species seem quite old, and divergence dating of Sumatran agamid phylogenies may corroborate estimated geologic events from before the Pleistocene such as those put forth by (Voris 2000).

### Phylogenetics

Phylogenetic analyses uncovered five distinct clades of *Dendragama*, among which are *D. australis*, *D. dioidema*, *D. schneideri* and two distinct clades of *D. boulengeri*, the first clade of *D. boulengeri* from Mount Kerinci and the second from near the type locality, Mount Marapi. These two clades are 5.0% pairwise genetically distant, which in many cases would constitute distinct species designations, if accompanied by readily identifiable morphological variation between populations. For example, Bradley and Baker (2009) show that sister species of mammals with readily distinguishable morphological characteristics typically had greater than 5.0% genetic variation in Cytochrome B sequences upon genetic evaluation later. *Dendragama boulengeri* populations examined in this study are on the edge of that genetic cutoff. Although means of some meristic and mensural characters differ between the Kerinci and Marapi populations of *D. boulengeri*, we did not find any fixed differences. Although geographically isolated from one another, we continue to recognize these two populations as *D. boulengeri*. However, we suspect that further studies may indeed identify these populations as distinct species.

These phylogenetic analyses further elucidate some of the patterns associated with cloud forest agamid lizard distributions in Sumatra. It seems that very few species have overlapping distributions and in contrast species, within *Dendragama*, species are distributed in a site-specific endemic pattern. Each species is distributed allopatrically with relatively small geographic ranges that match natural breaks in cloud forest habitat due to elevational changes. Furthermore, *Lophocalotes* is sister to the *Dendragama* group followed by insular *Pseudocalotes*, supporting Harvey et al. (2017).

### Conservation

The conservation status of species of *Dendragama* has not yet been assessed by the IUCN (http://www.iucnredlist.org/), particularly because of the lack of population data. However, it is clear that *Dendragama* inhabits isolated cloud forest patches and Sumatra’s rapid rates of deforestation are causing some of those patches to decline in size. Between 1900 and 2019 Sumatra’s lowland forests were nearly wiped out for agriculture and timber, and because of forest fires (Poor et al. 2019). Deforestation is extending into highland areas that encompass remaining patches of *D. schneideri* habitat. Many forested areas have already been diminished beyond macroecological tipping points (Nowosad et al. 2019) and the ecological ramifications could cascade throughout the remaining cloud forest habitat.

## Conclusions

The information from this paper contributes data on populations of *D. boulengeri* and *D. schneideri*, which we believe have small distributions, but are found in high abundance within those ranges. We estimated the latitudinal and longitudinal limits of *D. boulengeri* and *D. schneideri* (Fig. 1); however, we hypothesize that inventories of the rest of Sumatra’s mountains would yield an array of new species and would show that the true distributional limits of all *Dendragama* species are actually small. These data may be used towards conducting IUCN Red List status assessments in the future; however additional information is needed to provide proper assessments. Regardless of their current conservation status, it is clear that rapidly increasing anthropogenic pressures throughout Sumatra are likely to have a significant impact on all species of *Dendragama*. Shaney et al. (2016) provide examples of how cryptic Indonesian lineages may be lost before being described and cryptic species may be overharvested due to poor taxonomic evaluation. Thus, continuation of biological inventory will be important in agamid lizard discovery and conservation of Sumatra’s montane forest diversity in the near future. Given the rapid discovery of herpetofaunal diversity across Sumatra’s highlands (Iskandar 2006; Kurniati et al. 2009) it is likely that an array of new agamid lizard species remains undiscovered throughout the region (Shaney et al. 2016).

## Supplementary Material

XML Treatment for Dendragama schneideri

## Supplementary materials

Supplementary material 1 List of additional specimens examined for morphological work, museum IDs and species names Data type specimens examined https://binary.pensoft.net/file/476876This dataset is made available under the Open Database License (http://opendatacommons.org/licenses/odbl/1.0/). The Open Database License (ODbL) is a license agreement intended to allow users to freely share, modify, and use this Dataset while maintaining this same freedom for others, provided that the original source and author(s) are credited.Kyle J. Shaney, Michael B. Harvey, Amir Hamidy, Nia Kurniawan, Eric N. Smith

Supplementary material 2 Specimens included in phylogenetic assessment and GenBank accession numbers Data type genbank data https://binary.pensoft.net/file/476877This dataset is made available under the Open Database License (http://opendatacommons.org/licenses/odbl/1.0/). The Open Database License (ODbL) is a license agreement intended to allow users to freely share, modify, and use this Dataset while maintaining this same freedom for others, provided that the original source and author(s) are credited.Kyle J. Shaney, Michael B. Harvey, Amir Hamidy, Nia Kurniawan, Eric N. Smith
