# Supplementary material for: Phylogeny and biogeography of Sumatra´s cloud forest lizards of the genus Dendragama and status of Acanthosaura schneideri
Source: Zookeys. 2020 Nov 18;995:127–53. doi: 10.3897/zookeys.995.49355 (PMC7688618; doi:10.3897/zookeys.995.49355)
Supplement: Supplementary material 1 — List of additional specimens examined for morphological work, museum IDs and species names [file zookeys-995-127-s001.docx]

*Dendragama australis* (38): INDONESIA. SUMATERA SELATAN. An adult male (MZB

13786, Holotype) from trail up Gunung Dempo above Kampung Empat, Kabupaten Pagaralam, Provinsi Sumatera Selatan, Indonesia, 4.03744°S, 103.14526°E, 1953 m, seven males (MZB 13776, 13778, 13782, 13783; UTA 63420, 63421, 63423), four females (MZB 13777, 13779, 13781, UTA 63424), and four unsexed specimens (MZB 13780, UTA 63418, 63419, 63422) from Gunung Patah near Desa Segamit, Kabupaten Muara Enim, Provinsi Sumatera Selatan, Indonesia, 4.21–4.23°S, 103.41–103.42°E, 1742–2142 m, three males (MZB 13784, UTA 63427, 63428), two females (MZB 13785, 13787), and six unsexed specimens (MZB 13788, UTA 63425, 63426, 63429–63431) from Gunung Dempo above Kampung Empat, Kabupaten Pagaralam, Provinsi Sumatera Selatan, Indonesia, 4.04°S, 103.14– 103.17°E, 1764–2111 m. INDONESIA. BENGKULU. Three males (MZB 13789, 13790, UTA 63433) and one unsexed specimen (UTA 63432) from Bukit Daun, above desa Air Nipas, Rimbo Pengadang, Kabupaten Rejang Lebong, 3.36°S, 102.38° E, 1646–1728 m, five males (MZB 13791–13793; UTA 63434, 63435), one female (UTA 63436), and one unsexed specimen (UTA 63437) from Air Duku, Selupu Rejang, trail up Gunung Kambing from police academy near road to Gunung Kaba, Kabupaten Rejang Lebong, 3.39–3.40°S, 102.63–102.64°E, 1516– 1748 m.

*Dendragama boulengeri* (27): INDONESIA. SUMATERA BARAT: One male (MSNG 29936,

Lectotype) from “monte Singalang (Sumatra occidentale) ad un’ altezza di metri circa 2800” (traced to Gunung Singgalang, Kabupaten Agam, 0.38°S, 100.36°E), five paralectotypes, nine males including (BMNH 1946.8.13.15; ZMB 10155, 54503, MZB 13822, 13824, 43 13825; UTA 63463, 63466, 63469) and 10 females (MNHN 1889- 27; ZMB 54502, MZB 13819– 13821, 13823, 13826; UTA 63465, 63467) from near the type locality (above desa Beringin) 0.3752–0.3753°S, 100.363°E, 1376–1473 m, and seven unsexed specimens (MZB 13817, 13818, 13824; UTA 63461, 63462, 63464, 63468) from Aie Angek, Sepuluh Koto, Gunung Marapi, Kabupaten Tanah Datar, 0.395°S, 100.425°E, 1526–1553 m, nine specimens (UTA 62852–62870) from Gunung Kerinci, Jambi Province, 1.710°S, 101.253°E, 1400–1800 m.

*Dendragama dioidema* (43): INDONESIA. ACEH. An adult male (MZB 13814, Holotype) from Bukit Sama, Kampung Telege Atu, Kebayakan, Kabupaten Aceh Tengah, 4.66512° N, 96.80937° E, 1567 m, Three males (MZB 13794; UTA 63448, 63449), six females (MZB 13804, 13805; UTA 63438, 63440, 63441, 63446), and 15 unsexed specimens (MZB 13795–13803; UTA 63439, 63442–63445, 63447) from Kute Baru, Linge, along road from Takengon to Isaq, Kabupaten Aceh Tengah, 4.52875° N, 96.85316° E, 1827 m, one unsexed specimen (UTA 63450) from foot of Berni Terlong, near Desa Rambune, Kabupaten Bener Meriah, 4.7648° N, 96.80196° E, 1471 m, four male (MZB 13807, 13808, 13810; UTA 63452) and two unsexed specimens (MZB 13809; UTA 63453) from Beutong Ule, high point on Meulaboh-Takengon road, Kabupaten Nagan Raya, 4.38367° N, 96.51633° E, 1950 m, one male (MZB 13811), three females (UTA 63456–63458), and three unsexed specimens (MZB 13812, 13813; UTA 63455) from Bukit Sama, Kampung Telege Atu, Kebayakan, Kabupaten Aceh Tengah, 4.66512– 4.66583° N, 96.80627– 96.80937° E, one male (UTA 63460), one female (MZB 13815), and two unsexed specimens (MZB 13816; UTA 63459) from Hutan Timang Gajah, Gunung Burni Telong, Kabupaten Bener Meriah, 4.77122–4.77142° N, 96.80907– 96.81017° E, 1875–1957 m.

*Pseudocalotes brevipes* (10): LAO PDR. XE KONG; Kaleum District, Xe Sap National Biodiversity Conservation Area, near 16° 0410N, 106° 5845”E, 1200–1300 m (FMNH 258703). VIETNAM. VINH PHU; Tam Dao, Vinh Yen District (MVZ 224103–224106; 226486, 226487, 226489, 226490, 226494).

*Pseudocalotes cybelidermus* (15): INDONESIA. LAMPUNG: Montane forest above Ngarip, Lampung, Sumatra, Indonesia, 5.28°S, 104.56° E, 1376–1521 m (MZB 9766, 9769, 9800, UTA 60537–60539, 60552–6055 paratypes). SUMATERA SELATAN: Montane forest at Maura Dua, Remanan Jaya, Gunung Pesagi (locally known as Masagi), 4.91°S, 104.13°E, 1474–1643 m (MZB 9767 holotype; MZB 9650, 9760, 9799, UTA 60549–60551 paratypes).

*Pseudocalotes dringi*: MALAYSIA. PAHANG: “Gunung Tahan, 6500–7200 ft (1981–2194 m) elevation” (BMNH 1906.2.28.10 holotype). TERENGGANU: “Summit Ridge, 4000 ft (1219 m) elevation, Gunung Lawit, Terengganu, W-Malaysia” (BMNH 1974.4929 paratype).

*Pseudocalotes flavigula* (1): MALAYSIA. PAHANG; Gunnong Brinchang, Cameron Highlands,

1524–1829 m (FMNH 143903); “Cameron Highlands between 5,000 and 6,000 feet” (BMNH

1946.8.11.14 holotype).

*Pseudocalotes floweri* (2): CAMBODIA. KOH KONG; Cardamom Highlands Plateau. 0349359

Easting, 1325813 Northing, 1200 m (FMNH 270127). THAILAND. No other data (FMNH 114514), “Chantaboon” = Chantaburin (BMNH 1946.8.11.25 lectotype).

*Pseudocalotes guttalineatus* (16): INDONESIA. LAMPUNG: Montane forest above Ngarip, 5.28°S, 104.56° E, 1341–1521 m (MZB 9796 holotype; MZB 9652, 9765, 9792–9795, 9801, UTA 60536, 60540–60543, 60554 paratypes). SUMATERA SELATAN: Maura Dua, Remanan Jaya, Gunung Pesagi (localy known as Masagi), 4.91°S, 104.13°E, 1574–1643 m (UTA 60500–60501, paratypes).

*Pseudocalotes kakhienensis* (6): CHINA. YUNAN; Nujiang Prefecture, small village S of Gongshan, 27° 42' 13.7016" N, 98° 42' 10.1982" E, ca 1451 m (CAS 214907, 214940, 214949), Fugong County, Shiwuli, 27° 09’ 22.5” N, 98° 47’ 57.4” E (CAS 234454–234455), Gongshan County, vicinity of village S of Gongshan, 27° 42’ 13.1” N, 98° 42’ 10.6” E, 1437 m (CAS 242105).

*Pseudocalotes kingdonwardi* (11): BURMA. “Adung Valley, 7000ft, Burma-Tibet border” (BMNH 1946.8.11.17 holotype). CHINA. YUNAN; Nujiang Prefecture, Gongshan County; road between Kongdang and Bapo in Dulong Valley, 27° 49’ 33.9” N, 98° 19’ 31.7” E, 1478 m (CAS 241965), ca. 5 km N (by Dulong River) of Kongdang (CAS 241992, 241994, 241997), Dulong Valley, E of Kongdang (CAS 242015), Dulong Valley, 2 km N of Kongdang, W side of Dulong River (CAS 242020), Dulong Valley, road from Bapo N toward Kongdang, 27° 45’ 29.9” N, 98° 20’ 52.8” E, 1357 m (CAS 242628), Dulong Valley, Kongdang, 27° 50’ 28.4” N, 98° 19’ 45.4” E, 1450 m (CAS 242653), Dulong Valley, Kongdang, 27° 52’ 07.7” N, 98° 20’ 09.4” E, 1451 m (CAS 242674), Dulong Valley, Kongdang, 27° 51’ 44.0” N, 98° 20’ 04.3” E (CAS 242675), Dulong Valley, Kongdang, 27° 52’ 07.2” N, 98° 20’ 09.8” E, 1450 m (CAS 242676).

*Pseudocalotes microlepis* (2): LAO PDR. Phong Saly (FMNH 14499). VIETNAM. BAC THAI; Ba Be Lake National Park, cave area, ca. 1.5 km E of guest house, 22° 24’ 1” N, 105° 37’ 54” E. THAILAND. “Plapoo Tenasserim” (BMNH 1946.8.11.21).

*Pseudocalotes poilani* (2): LAO PDR. CHAMPASAK; Pakxong District, Dong Hua Sao National Biodiversity Conservation Area, near Ban Nongluang Village, near 15° 0470N, 106° 1203”E, 1100 m (FMNH 258704), Pakxong District, Dong Hua Sao National Biodiversity Conservation Area, Bolaven Plateau, near 15° 0355N, 106° 1303”E, 1200 m (FMNH 258710).

*Pseudocalotes rhammanotus* (1). INDONESIA. LAMPUNG: montane forest along the ridge of a mountain south of Danau Ranau (= Lake Ranau), 4.9394° S, 103.85292° E, 1237 m (MZB 10804, holotype).

*Pseudocalotes tympanistriga* (16): INDONESIA. JAWA BARAT. Cibodas Botanical Garden, 6.74181° S, 107.0061° E (UTA 60544–60548); Gunung Tilu, 7.15601° S, 107.52309° E, 1389–1647 m (UTA 63094–63096); road from S coast of Java to Gunung Patuha, 7.24716° S, 107.35696° E, 1156 m (UTA R 63097); road from S coast of Java to Gunung Patuha, 7.24711° S, 107.35722° E, 1183 m (UTA R 63098–63099); Gunung Puntang. Gibbon Research Center, 7.11828° S, 107.60493° E, 1393–1468 m (UTA 63100–63101); Gunung Waringin, 7.15603° S, 107.49647° E, 1583–1607 m (UTA 63102–63103).

NO OTHER DATA: “Java” (ZMB 689 holotype).
